# Risk Factors and Outcomes of Protein-Calorie Malnutrition in Chronic Heart Failure Patients Undergoing Elective Cardiac Surgery

**DOI:** 10.7759/cureus.30378

**Published:** 2022-10-17

**Authors:** Talha Mubashir, Julius Balogh, Emily Breland, Dustin Rumpel, Maham A Waheed, Hongyin Lai, Charles A Napolitano

**Affiliations:** 1 Department of Anesthesiology, University of Arkansas for Medical Sciences, Little Rock, USA; 2 Department of Internal Medicine, Maimonides Medical Center, New York City, USA; 3 Department of Biostatistics, University of Texas at Houston, Houston, USA

**Keywords:** adults, valve replacement surgery, coronary artery bypass graft, protein-calorie malnutrition, chronic heart failure

## Abstract

Introduction: Chronic heart failure (CHF) patients are often malnourished. Our aim was to determine the effect of protein-calorie malnutrition (PCM) on in-hospital outcomes in CHF patients following elective cardiac surgery and to identify risk factors for PCM in this patient population.

Methods: A retrospective analysis of the National Inpatient Sample (NIS) database was conducted from 2016 to 2018. In-hospital outcomes in adult patients with CHF undergoing elective coronary artery bypass graft (CABG) with cardiopulmonary bypass-assist or cardiac valve replacement surgeries were analyzed. Propensity-score matching was used to match CHF patients with and without PCM and followed by logistic regression analysis. A multivariate logistic regression model was used to identify the risk factors associated with PCM in this population.

Results: In total 25,940 CHF patients were identified, of which 6,271 underwent elective CABG and 19,669 underwent valve replacement surgeries. The prevalence of PCM in CHF patients undergoing CABG and valve replacement was 3.9% and 2.9%, respectively. CHF patients with PCM had significantly higher risk of in-hospital mortality, post-operative cardiac and gastrointestinal complications compared to CHF patients without PCM. The mean hospital length of stay was twice as high in the PCM group (mean days: 18.6 vs 9.9). Female gender, Black race (vs White race), a high Charlson Comorbidity Index, Medicare/Medicaid insurance status (vs private insurance), and CHF (systolic and combined systolic and diastolic) were independently associated with significantly higher risk of PCM diagnosis.

Conclusions: CHF patients with PCM who undergo elective CABG or valve replacement surgeries are at a significantly higher risk of mortality, post-operative cardiac and gastrointestinal complications, and increased duration of hospital stay compared to those without PCM. Future prospective studies should assess the CHF patients who are at a higher risk of PCM and whether correcting pre-operative nutrition in this surgical population can improve outcomes following cardiac surgery.

## Introduction

Among patients undergoing cardiac surgery, the prevalence of malnutrition may be 18-40% at the time of surgery [1-3]. Protein-calorie malnutrition (PCM) develops from insufficient protein and/or caloric (i.e., energy) intake. This can result in unintended weight loss, including loss of muscle mass or loss of subcutaneous fat, tissue fluid accumulation (e.g., low oncotic pressures), and diminished physical function [4]. In addition, PCM has significant impact on multiple systems of the body including weakness of skeletal muscles, reduced heart muscle mass, impaired wound healing, immune deficiency, and hypothermia [5]. Previous research has indicated that PCM leads to an increased risk of morbidity and mortality, hospital length of stay (LOS), and complications in cardiac surgery patients [6]. Current data on in-hospital outcomes for chronic heart failure (CHF) patients with PCM undergoing cardiac surgery is limited.

In the presence of CHF, the intestine and the heart develop a bidirectional relationship, the clinical manifestation of which is cardiointestinal syndrome (CIS) [7]. Accordingly, advanced heart failure (HF) is commonly associated with gastrointestinal (GI) symptoms, including abdominal pain, anorexia, and protein-losing enteropathy. Conditions that affect both these systems, such as PCM, stand out as under-explored comorbidities that may affect cardiac surgery outcomes. The systemic inflammation and stress of cardiac surgery can affect PCM patients more profoundly than their well-nourished counterparts whose morbidity and mortality after surgery are significantly lower [8]. The objective of this retrospective cohort analysis was to investigate in-hospital outcomes of CHF patients with and without PCM following elective cardiac surgery. Additionally, we sought to identify risk factors associated with PCM in patients undergoing elective cardiac surgery. This article was previously presented as a meeting abstract at the 2022 Society of Cardiac Anesthesiology Meeting on May 15, 2022.

## Materials and methods

Data collection

A retrospective population-based analysis utilizing 2016-2018 National Inpatient Sample (NIS) data was performed [9]. From 2016 onward, the NIS data uses the International Classification of Diseases 10th edition, Clinical Modification/Procedure Coding System (ICD-10-CM/PCS). The ICD-10-CM/PCM codes were used to conduct the analysis. The NIS is one of several nationwide databases part of the Healthcare Cost and Utilization Project (HCUP), Agency for Healthcare Research and Quality. The NIS contains de-identified patient information and conforms to Health Insurance Portability and Accountability Act (HIPAA) guidelines. Hence, written informed consent was not required per the exemption by the institutional review board (IRB). The authors adhered to the HCUP Data Use Agreement. Strengthening the reporting of observational studies in epidemiology (STROBE) checklist for cohort studies was followed.

The details of the diagnostic and procedure codes used to build the study population are presented in Appendix 1. CHF codes included I50.22, I50.23, I50.32, I50.33, I50.42, and I50.43, while PCM was coded using E43, E44, and E46. Information on demographic characteristics (i.e., age, gender, ethnicity), primary insurance status (i.e., Medicare/Medicaid, private, self-pay), and patient comorbidities were extracted. The Charlson Comorbidity Index (CCI) was calculated for each patient and categorized into the following groups: no comorbidity (0), mild comorbidity (1-2), moderate comorbidity (3-4), and severe comorbidity (≥5) [10]. In addition, data were extracted on commonly associated comorbidities in the cardiac surgical population including hypertension, diabetes mellitus (DM), obesity, dyslipidemia, chronic obstructive pulmonary disease (COPD), end-stage renal disease (ESRD), chronic kidney disease (CKD) stages 2-4, prior myocardial infarction (MI), known coronary artery disease (CAD), chronic pulmonary hypertension, peripheral vascular disease (PVD), and history of ischemic cerebrovascular accidents (CVA).

Study cohort

Adult patients (age ≥18 years) that underwent coronary artery bypass graft (CABG) surgery with cardiopulmonary bypass-assist (CPB) or valve replacement surgeries (i.e., aortic, pulmonic, mitral, or tricuspid) during elective hospital admission were included. Patients undergoing elective CABGs or valve replacement surgeries were categorized into CHF and no heart failure (HF) groups. If patients did not have an acute or chronic HF diagnosis code, they were categorized as having no HF. Additionally, among the CHF group, those with a diagnosis of PCM were identified using the Centers for Medicare and Medicaid Services (CMS) definition. The CMS has defined PCM as any two of the following: (1) muscle wasting and loss of subcutaneous fat, (2) <50-75% of normal nutritional intake over >1-2 weeks, (3) reduced functional capacity, and (4) weight loss [11].

Exclusion criteria were as follows: (1) age <18 years, (2) isolated acute HF, (3) concomitant CABG and valve replacement surgeries during same or different hospitalizations, (4) history or current hospitalization for cardiac transplant, (5) malignancy, (6) intestinal malabsorption disorders (e.g., celiac disease, tropical sprue, etc.), and (7) rare nutritional disorders (i.e., kwashiorkor and marasmus).

Outcomes

The primary outcome was the risk of in-hospital mortality in CHF patients with pre-existing PCM vs no PCM following elective cardiac surgery (i.e., CABG or valve replacement), while secondary outcomes included hospital LOS and the risk of acute cardiac and GI complications. The composite cardiac complications included - acute MI, cardiogenic shock, cardiac arrest, ventricular tachycardia (VT)/ventricular fibrillation (VF), cardiac tamponade, and intra-aortic balloon pump (IABP) use. The composite GI complications included - GI bleeding, intestinal ischemia or infarction, and acute hepatic failure. Additional secondary outcomes included assessing the risk factors for a PCM diagnosis in patients undergoing cardiac surgery to identify vulnerable populations.

Statistical analysis

For assessing differences in categorical variables a Chi-square test was used, while for continuous variables a two-sample t-test was used for assessing the mean difference. Data are presented as frequency (%) or mean (standard deviation {SD}) for categorical and continuous variables, respectively. In the assessment of the primary and secondary outcomes between PCM and non-PCM CHF groups, a 1:6 propensity-score matching (PSM) was utilized to minimize the probability of selection bias. Matching factors included demographic characteristics (e.g., age, gender, race), comorbidities (i.e., CCI), and insurance status. Following PSM, logistic regression was used to assess the effects of PCM in CHF patients undergoing cardiac surgery on the primary and secondary outcomes. The categorical outcomes are reported as odds ratio (OR) with a 95% confidence interval (CI) and a p-value (significant if <0.05). Finally, for the identification of potential risk factors for PCM diagnosis in patients undergoing elective cardiac surgery, a multivariate logistic regression model was utilized to calculate the OR. Statistical software for data sciences, STATA 14 (College Station, TX: StataCorp LLC), was used for data extraction and analysis.

## Results

Outcomes

During the 2016 to 2018 study period, 43,253 elective CABGs and 42,162 elective valve replacement surgeries were identified of whom 6,271 (14.5%) and 19,669 (46.7%) patients had CHF, respectively. Compared to patients without HF, CHF patients undergoing cardiac surgery were significantly older (73.5±11.8 vs 66.8±11.7, p<0.001), had a greater proportion of females (40.9% vs 29.6%, p<0.001), a higher CCI index (moderate-to-severe CCI: 59.5% vs 23%, p<0.001) and had a higher frequency of Medicare/Medicaid insurance status (81% vs 64%, p<0.001). Table 1 depicts the baseline characteristics and comorbidities of patients with CHF and those without HF.

**Table 1:** Unadjusted baseline characteristics comparing chronic heart failure (CHF) vs no heart failure (HF) in patients undergoing cardiac surgeries from the NIS database between 2016 and 2018 in the United States. CABG: coronary artery bypass graft; CAD: coronary artery disease; CCI: Charlson Comorbidity Index; CHF: chronic heart failure; COPD: chronic obstructive pulmonary disease; CKD: chronic kidney disease; CVA: cerebrovascular accident; HF: heart failure; PCM: protein-calorie malnutrition; PVD: peripheral vascular disease; SD: standard deviation

Variables	CABG (N=43,253)		Valve replacement (N=42,162)	
	Chronic HF (n=6,271; 14.5%)	No HF (n=36,982; 85.5%)	Chronic HF (n=19,669; 46.7%)	No HF (n=22,493; 53.3%)
Age, mean (SD)	66.7 (9.8)	65.9 (9.5)	75.7 (11.6)	68.2 (14.5)
Gender, n (%)				
Male	4,745 (75.7)	28,842 (78)	10,582 (53.8)	13,026 (57.9)
Female	1,526 (24.3)	8,134 (22)	9,085 (46.2)	9,460 (42.1)
Race, n (%)				
White	4,500 (71.8)	28,968 (78.3)	16,091 (81.8)	18,431 (82)
Black	542 (8.6)	1,915 (5.2)	1,102 (5.6)	885 (3.9)
Hispanic	459 (7.3)	2,365 (6.4)	883 (4.5)	1,311 (5.8)
Other/missing	770 (12.3)	3,734 (10.1)	1,593 (8.1)	1,866 (8.3)
CCI, n (%)				
0	0	8,419 (22.7)	0	7,294 (32.4)
Mild (1-2)	2,017 (32.2)	18,972 (51.3)	8,489 (43.2)	11,145 (49.5)
Moderate (3-4)	2,328 (37.1)	7,007 (19)	6,768 (34.4)	3,109 (13.8)
Severe (≥5)	1,924 (30.7)	2,586 (7)	4,397 (22.4)	960 (4.3)
Primary payer, n (%)				
Medicare/Medicaid	4,339 (69.2)	22,719 (61.4)	16,670 (84.8)	15,370 (68.3)
Private insurance	1,673 (26.7)	12,742 (34.5)	2,544 (12.9)	6,440 (28.6)
Self-pay/other/missing	259 (4.1)	1,521 (4.1)	455 (2.3)	683 (3.0)
Comorbidities, n (%)				
Hypertension	1,060 (16.9)	25,996 (70.3)	2,986 (15.2)	13,761 (61.2)
Diabetes mellitus	3,505 (55.9)	16,774 (45.4)	7,004 (35.6)	5,749 (25.6)
Dyslipidemia	4,920 (78.5)	30,955 (83.7)	13,349 (67.9)	13,961 (62.1)
COPD	1,495 (23.8)	5,539 (15)	4,718 (24)	3,005 (13.4)
End-stage renal disease (stage 5)	365 (32.5)	758 (2.1)	649 (3.3)	327 (1.4)
CKD stage 2, 3 and 4	322 (5.1)	870 (2.3)	1,080 (5.5)	451 (2)
Obesity	1,922 (30.6)	9,569 (25.9)	4,267 (21.7)	4,632 (20.6)
Prior myocardial infarction	1,790 (28.5)	6,865 (18.6)	2,379 (12.1)	1,294 (5.8)
Known CAD	6,271 (100)	36,982 (100)	12,289 (62.5)	9,473 (42.1)
Chronic pulmonary hypertension	735 (11.7)	822 (2.2)	4,138 (21)	2,238 (9.9)
PVD	132 (2.1)	457 (1.2)	403 (2.1)	232 (1.0)
Ischemic CVA	590 (9.4)	2,929 (7.9)	1,420 (7.2)	1,256 (5.6)
PCM	247 (3.9)	469 (1.3)	576 (2.9)	350 (1.6)

Overall, 1,642 cardiac surgery patients had a PCM diagnosis with an overall prevalence of 1.9%. Compared to patients without HF, CHF patients undergoing CABG (3.9% vs 1.3%, p<0.001) and valve replacement surgeries (2.9% vs 1.6%, p<0.001) had higher frequency of PCM. The adjusted risk of having PCM was significantly higher in CHF patients undergoing CABG (OR 2.06, 95% CI 1.74-2.45, p<0.001) and valve replacement (OR 1.46, 95% CI 1.25-1.72, p<0.001) surgeries when compared to patients without HF. Following propensity-score matching, 247 (14.3%) CHF patients with PCM were identified who underwent CABG, while 574 (14.2%) CHF patients with PCM were identified who underwent valve replacement surgery (Appendix 2).

The risk of in-hospital mortality was significantly higher in CHF patients with PCM following CABG (OR 5.0, 95% CI 2.66-9.42, p<0.001) and valve replacement (OR 3.5, 95% CI 2.30-5.23, p<0.001) surgery, compared to those without PCM. Table 2 shows the primary and secondary in-hospital outcomes of CHF patients with and without pre-existing PCM undergoing cardiac surgery following propensity-score matching. The composite risk of cardiac (OR 2.9, 95% CI 2.55-3.31, p<0.001) and GI (OR 8.4, 95% CI 6.17-11.5, p<0.001) complications were significantly higher in the PCM group (vs no PCM) undergoing cardiac surgery.

**Table 2:** Propensity-score matched models (1:6) to measure odds ratio (OR) of in-hospital outcomes following elective cardiac surgeries in chronic heart failure patients comparing protein-calorie malnutrition vs no protein-calorie malnutrition. CABG: coronary artery bypass graft; CHF: chronic heart failure; CI: confidence interval; IABP: intra-aortic balloon pump; LOS: length of stay; MI: myocardial infarction; OR: odds ratio; PCM: protein-calorie malnutrition; SD: standard deviation; VT/VF: ventricular tachycardia/ventricular fibrillation

Outcomes	All cardiac surgeries		CABG		Valve replacement	
	OR (95% CI)	p-value	OR (95% CI)	p-value	OR (95% CI)	p-value
In-hospital mortality						
Overall CHF	3.6 (2.84-4.81)	<0.001	5.0 (2.66-9.42)	<0.001	3.5 (2.30-5.23)	<0.001
Cardiac complications	2.9 (2.55-3.31)	<0.001	2.43 (1.85-3.20)	<0.001	3.3 (2.65-4.06)	<0.001
Acute MI	1.5 (1.14-1.97)	0.004	1.2 (0.82-1.72)	0.355	2.6 (1.33-4.91)	0.005
Cardiogenic shock	4.0 (3.41-4.88)	<0.001	3.6 (2.53-5.20)	<0.001	4.3 (3.20-5.78)	<0.001
Cardiac arrest	3.7 (2.63-5.33)	<0.001	5.1 (2.42-10.7)	<0.001	3.4 (1.94-6.06)	<0.001
VT/VF	2.1 (1.82-2.70)	<0.001	2.7 (1.79-4.02)	<0.001	2.1 (1.52-2.88)	<0.001
Cardiac tamponade	3.6 (2.43-5.40)	<0.001	1.8 (0.49-6.62)	0.370	3.9 (2.17-7.19)	<0.001
IABP	3.2 (2.59-4.12)	<0.001	2.5 (1.72-3.73)	<0.001	4.0 (2.64-6.14)	<0.001
Gastrointestinal complications	8.4 (6.17-11.5)	<0.001	10.0 (5.41-18.4)	<0.001	8.0 (4.77-13.3)	<0.001
Gastrointestinal bleeding	5.8 (3.37-10.2)	<0.001	15.3 (2.94-79.2)	0.001	5.1 (2.18-11.8)	<0.001
Intestinal ischemia or infarction	20.1 (5.54-73.3)	<0.001	12.1 (1.1-133.8)	0.042	24.2 (2.70-216.6)	0.004
Acute hepatic failure	9.8 (6.72-14.4)	<0.001	8.5 (4.38-16.6)	<0.001	10.7 (5.50-20.8)	<0.001
Hospital LOS (days), mean (SD), median						
PCM	18.6 (15.6), 14	<0.001	18.6 (15.7), 14	<0.001	12.9 (13.4), 8	<0.001
No PCM	9.9 (9.5), 8		9.9 (9.6), 8		5.7 (5.8), 4	

CHF patients with PCM (vs no PCM) undergoing CABG had significantly higher risk of cardiogenic shock (OR 3.6, 95% CI 2.53-5.20, p<0.001), cardiac arrest (OR 5.1, 95% CI 2.42-10.7, p<0.001), VT/VF (OR 2.7, 95% CI 1.79-4.02, p<0.001), use of intra-aortic balloon pump (IABP) (OR 2.5, 95% CI 1.72-3.73, p<0.001), GI bleeding (OR 15.3, 95% CI 2.94-79.2, p=0.001), intestinal ischemia/infarction (OR 12.1, 95% CI 1.1-133.8, p=0.042), and acute hepatic failure (OR 8.5, 95% CI 4.38-16.6, p<0.001). CHF patients with PCM (vs no PCM) undergoing valve replacement surgery had significantly higher risk of acute MI (OR 2.6, 95% CI 1.33-4.91, p=0.005), cardiogenic shock (OR 4.3, 95% CI 3.20-5.78, p<0.001), cardiac arrest (OR 3.4, 95% CI 1.94-6.06, p<0.001), VT/VF (OR 2.1, 95% CI 1.52-2.88, p<0.001), cardiac tamponade (OR 3.9, 95% CI 2.17-7.19, p<0.001), IABP use (OR 4.0, 95% CI 2.64-6.14, p<0.001), GI bleeding (OR 5.1, 95% CI 2.18-11.8, p<0.001), intestinal ischemia/infarction (OR 24.2, 95% CI 2.70-216.6, p=0.004), and acute hepatic failure (OR 10.7, 95% CI 5.50-20.8, p<0.001).

After excluding CHF patients that died during hospitalization, the length of hospital stay in patients with PCM was significantly longer following CABG (median 14 days vs eight days, p<0.001) and valve replacement (median eight days vs four days, p<0.001), compared to patients without PCM (Table 2).

Risk factors for protein-calorie malnutrition

Older age (OR 1.02, 95% CI 1.02-1.04, p<0.001), female gender (OR 1.37, 95% CI 1.16-1.61, p<0.001), and a higher CCI (mild CCI: OR 2.04 vs moderate CCI: 3.20 vs severe CCI: 4.80, p<0.001) were associated with an increased risk of PCM in patients undergoing CABG. Table 3 depicts the risk factors for PCM diagnosis in patients undergoing elective CABG and valve replacement surgeries. In CHF patients undergoing CABG surgery, the risk of PCM was significantly higher in patients with systolic CHF (OR 2.20, 95% CI 1.79-2.70, p<0.001), followed by combined systolic and diastolic CHF (OR 2.09, 95% CI 1.53-2.86, p<0.001) and isolated diastolic CHF (OR 1.76, 95% CI 1.34-2.33, p<0.001) when compared to CABG patients without underlying HF. Moreover, CABG patients who had private insurance had a significantly lower risk of PCM (OR 0.75, 95% CI 0.61-0.93, p=0.008) compared to those on Medicare/Medicaid, while Black race was associated with a higher risk of PCM (OR 1.31, 95% CI 0.99-1.72, p=0.053) compared to White race, albeit not statistically significant. However, in the overall cardiac surgical population, Black patients were significantly more likely to have PCM compared to White patients (OR 1.24, 95% CI 1.03-1.50, p=0.024).

The risk of PCM was significantly higher in cardiac valve replacement surgical patients who were females (OR 1.24, 95% CI 1.09-1.42, p=0.001) and who had a high CCI (mild CCI: OR 1.46 vs moderate CCI: 2.42 vs severe CCI: 2.81, p<0.001). In CHF patients undergoing valve replacement surgery, the risk of PCM was significantly higher in patients with systolic CHF (OR 2.04, 95% CI 1.66-2.49, p<0.001) and combined systolic and diastolic CHF (OR 1.95, 95% CI 1.57-2.42, p<0.001), while there was no difference in patients with diastolic CHF (OR 1.07, 95% CI 0.89-1.28, p=0.460) when compared to patients without HF. Finally, older age (OR 0.99, 95% CI 0.98-0.99, p<0.001) and Hispanic (OR 0.65, 95% CI 0.46-0.92, p=0.016) patients undergoing valve replacement surgery were at a significantly lower risk of PCM compared to White race. For every one-year increase in age among the surgical valve replacement group, the risk of PCM decreased by 1% (Table 3).

**Table 3:** Adjusted predictors for the odds ratio (OR) of protein-calorie malnutrition diagnosis in patients undergoing elective cardiac surgery. CABG: coronary artery bypass graft; CCI: Charlson Comorbidity Index; CHF: chronic heart failure; CI: confidence interval; OR: odds ratio; PCM: protein-calorie malnutrition; SD: standard deviation

Variables	All cardiac surgeries		CABG		Valve replacement	
	OR (95% CI)	p-value	OR (95% CI)	p-value	OR (95% CI)	p-value
Age (per one-year increase)	1.00 (0.99-1.00)	0.390	1.02 (1.02-1.04)	<0.001	0.99 (0.98-0.99)	<0.001
Female	1.31 (1.18-1.45)	<0.001	1.37 (1.16-1.61)	<0.001	1.24 (1.09-1.42)	0.001
Race (ref. White race)						
Black	1.24 (1.03-1.50)	0.024	1.31 (0.99-1.72)	0.053	1.18 (0.91-1.54)	0.201
Hispanic	0.83 (0.66-1.04)	0.104	1.03 (0.77-1.39)	0.826	0.65 (0.46-0.92)	0.016
CCI (ref. no comorbidity)						
Mild CCI (1-2)	1.61 (1.32-1.96)	<0.001	2.04 (1.48-2.79)	<0.001	1.46 (1.13-1.89)	0.004
Moderate CCI (3-4)	2.55 (2.07-3.14)	<0.001	3.20 (2.31-4.45)	<0.001	2.42 (1.83-3.19)	<0.001
Severe CCI (>5)	3.34 (2.68-4.16)	<0.001	4.80 (3.41-6.76)	<0.001	2.81 (2.08-3.79)	<0.001
Primary Payer (ref. Medicare/Medicaid)						
Private insurance	0.74 (0.65-0.86)	<0.001	0.75 (0.61-0.93)	0.008	0.84 (0.69-1.03)	0.093
CHF (ref. no HF)						
Diastolic CHF	1.18 (1.02-1.36)	0.024	1.76 (1.34-2.33)	<0.001	1.07 (0.89-1.28)	0.460
Systolic CHF	2.05 (1.73-2.42)	<0.001	2.20 (1.79-2.70)	<0.001	2.04 (1.66-2.49)	<0.001
Combined CHF	2.15 (1.86-2.48)	<0.001	2.09 (1.53-2.86)	<0.001	1.95 (1.57-2.42)	<0.001

## Discussion

In CHF patients undergoing elective cardiac surgeries, pre-existing PCM is associated with significantly higher inpatient mortality and post-operative cardiac (i.e., cardiac arrest, cardiogenic shock, VT/VF, cardiac tamponade, use of IABP) and GI (i.e., GI bleeding, intestinal ischemia or infarction, and acute hepatic failure) complications. These findings support previously reported poorer outcomes (e.g., post-operative complications, mortality) in patients with PCM undergoing esophageal [12] and lung surgery [13], percutaneous coronary intervention [14], and transcatheter aortic valve replacement procedures [15,16]. In our cohort, patients with CHF (vs no HF) were more likely to be suffering from PCM. In addition, in CHF patients, PCM was associated with twice as longer hospitalization compared to patients without PCM. This is consistent with our current understanding of the relationship between PCM and multiple organ systems, and the downstream cascade induced by cardiac surgery and cardiopulmonary bypass.

PCM is a complex disease process involving nearly every organ system in the body. The hallmark of PCM is inadequate protein or energy intake, which leads to a chronic systemic inflammatory state termed cytokine-induced malnutrition [5]. The downstream effect of this chronic inflammatory state is a negative feedback loop, which further exacerbates the problem. Sarcopenia develops as the body adapts to this chronic state of malnutrition by reducing the basal metabolic rate and shifting muscle protein from cardiac and respiratory muscles to central lean tissues (liver, GI tract, renal, hematologic, and immune systems) [5]. Cardiac cachexia can ensue following the development of advancing HF and wasting syndrome may be present in over 20% of hospitalized HF patients [17]. In this study, CHF patients undergoing cardiac surgeries were twice as likely to have PCM compared to those without HF. Impairments of systolic cardiac function tend to have higher prevalence of nutritional derangements, although patients with diastolic dysfunction can also develop malnutrition, which is consistent with our findings [18].

The multisystem nature of PCM and resulting pathophysiology, in the context of CHF, contribute to increased mortality, complications, and hospital LOS for cardiac surgery patients [17,19,20]. GI hypoperfusion and bowel wall edema from inadequate splanchnic perfusion in HF predispose to bacterial translocation and endotoxemia, triggering release of cytokines and other pro-inflammatory mediators [7,21]. These circulating cytokines exert direct toxic effects on the heart, leading to depressed myocardial contractility and cardiogenic shock (and increased use of IABP), as well as an increased incidence of arrhythmias that can result in cardiac arrest. Hence, cardiointestinal syndrome (CIS) from decompensated HF leads to a bidirectional positive feedback loop that perpetuates cardiac impairment and worsens PCM. Furthermore, the increased gut permeability and right-sided filling pressures (e.g., high venous pressures) from advancing HF commonly lead to GI tract endothelial dysfunction, intestinal edema and ischemia/infarction, hepatic congestion and dysfunction (e.g., sinusoidal edema that limits oxygen diffusion to hepatocytes), resulting in an increased incidence of GI complications [7]. This is consistent with our findings that showed CHF patients who had PCM before undergoing cardiac surgery had higher risk of cardiac and GI complications and had longer hospital LOS compared to CHF patients without PCM. The increased long-term mortality observed in hospitalized HF patients with malnutrition may be partially attributed to the increase in cardiac and GI complications as observed in our study population [19,20].

Cardiac surgery and CPB can further exacerbate the aforementioned derangements [16]. Surgical trauma activates neutrophils, endothelial cells, platelets, and pro-inflammatory mediators, while CPB initiates an inflammatory response and ischemia-reperfusion injury [22]. Ultimately, this results in increased oxidative stress and systemic inflammation, leading to acute and persistent organ injury and dysfunction. As this study showed CHF patients with PCM undergoing cardiac surgery are susceptible to a higher incidence of complications and prolonged hospital stays.

This study also showed that certain subgroups of patients appear to have a higher risk of PCM diagnosis pre-operatively. For instance, compared to those with private insurance, Medicare and Medicaid patients have a higher incidence of PCM. Among other factors, this might be due to poor dietary intake in this population. Females also show a higher incidence of PCM diagnosis. Smaller body habitus and decreased protein reserve likely predispose females to PCM compared to their male counterparts. Systolic and combined (systolic and diastolic) HF patients are nearly twice as likely to be diagnosed with PCM. This finding is likely the result of the body’s adaptation to inadequate protein intake. An intra-cellular shift of muscle proteins from the cardiac and respiratory systems to the liver, GI tract, kidney, hematologic, and immune systems results in cardiac cachexia. Cardiac cachexia is more detrimental to the patient with systolic failure (or combined systolic and diastolic) than diastolic failure alone, as the failing heart is left unable to adequately contract. Lastly, increasing age places patients at increased risk of PCM [23]. These patients not only have a reduction in lean tissue mass and metabolic reserves but also decreased organ function and ability to tolerate oxidative stress [23]. Interestingly, elderly patients undergoing only valve surgery were not associated with an increased risk for PCM, which may be due to the increased therapeutic use of transcatheter approaches in this patient population.

Increased comorbid conditions, substantial surgical insult, and use of CPB make the post-operative cardiac patient vulnerable to complications. Pre-operative identification and correction of PCM in this patient population is prudent. The Malnutrition Universal Screening Tool has been shown to predict mortality in cardiovascular surgery patients. The use of such screening tools should be assessed in CHF patients undergoing cardiac surgery in future studies to identify patients who are malnourished or at risk [1]. It has been demonstrated that intense nutritional support is beneficial in certain subsets of patients at high risk for PCM [24]. In fact, the European Society for Clinical Nutrition and Metabolism guidelines recommend medical nutrition therapy for seven to 14 days pre-operatively for severely malnourished patients, even if this delays surgery [25]. Compared to CHF patients with PCM, those without PCM are likely to have decreased cytokine-induced malnutrition, thereby attenuating the inflammatory response and downstream effects. Optimizing metabolic reserves prior to operation likely confers a similar benefit. Physicians, including anesthesiologists involved in the peri-operative assessment and optimization of patients for cardiac surgery, should focus on the early identification and treatment of PCM utilizing the expertise of a registered dietitian.

This study is limited by its retrospective design and reliance upon a documented diagnosis of PCM. PCM may therefore be underdiagnosed or underreported. In addition, multiple scoring systems exist for identification of PCM, and there is no standardized definition of PCM diagnosis. Hence, the generalizability of these results is challenging across this patient population and across centers in the United States. Additionally, due to the retrospective nature and the use of a non-granular database, the inclusion of a variety of disease-specific variables, and other intra-operative factors that could impact the outcomes of this study are omitted. Moreover, retrospective studies that examine associations based on administrative datasets are hypothesis-generating rather than proof of relationships. Hence, prospective studies are needed to confirm the findings of this study.

## Conclusions

CHF patients undergoing elective CABG or valve replacement surgeries are twice as likely to have pre-existing PCM compared to patients without HF. PCM is more likely to be present in patients with systolic or combined CHF (systolic and diastolic CHF) patients. Following elective cardiac surgery, CHF patients with PCM (vs without PCM) have higher hospital mortality, post-operative cardiac and GI complications, and longer duration of hospital stay. Nutritional status in this surgical population should be assessed using standardized definitions by prospective studies to determine whether timely correction pre-operatively improves prognosis. This appears most critical for individuals who are females, Black, with multiple comorbidities, on governmental insurance plans such as Medicare/Medicaid, and with systolic CHF. Hence, future prospective studies on CHF patients undergoing cardiac surgeries need to identify the type of nutritional problem and assess whether implementation of a personalized dietary plan prior to cardiac surgery can improve outcomes.

## Appendices

Appendix 1 

**Table 4:** ICD-10 diagnosis and procedure codes. ICD-10: International Classification of Diseases 10th Revision; CABG: coronary artery bypass graft; IABP: intra-aortic balloon pump

In-hospital complications	
I21	Acute myocardial infarction
I46	Cardiac arrest
R57.0	Cardiogenic shock
I47.2, I49.01	Ventricular tachycardia or ventricular fibrillation
I31.4	Cardiac tamponade
5A02210, 5A02110	IABP
K92.0, K92.1, K92.2, K91.841, K91.871	Gastrointestinal bleeding
K55.0	Intestinal ischemia or infarction
K72.0, K91.82	Acute hepatic failure
Comorbid Diagnoses and Procedures	
021008, 021009, 02100A, 02100J, 02100K, 02100Z, 021108, 021109, 02110A, 02110J, 02110K, 02110Z, 021208, 021209, 02120A, 02120J, 02120K, 02120Z, 021308, 021309, 02130A, 02130J, 02130K, 02130Z	CABG surgery (open)
02RF0, 02RF3, 02RG0, 02RG3, 02RH0, 02RH3, 02RJ0, 02RJ3	Valve replacement surgery
I50.22, I50.23, I50.32, I50.33, I50.42, I50.43	Chronic heart failure
I50.32, I50.33	Diastolic chronic heart failure
I50.22, I50.23	Systolic chronic heart failure
I50.42, I50.43	Combined chronic heart failure
E43, E44, E46	Protein-calorie malnutrition
I10, I15	Hypertension
E08, E09, E10, E11, E13	Diabetes mellitus
E78	Dyslipidemia
J41, J42, J43, J44	Chronic obstructive pulmonary disease
I12.0, I13.11, I13.2, N18.5, N18.6	End-stage renal disease (stage 5)
N18.2, N18.3, N18.4	Chronic kidney disease stage 2, 3, and 4
E66.0, E66.2, E66.8, E66.9	Obesity
I25.2	Prior myocardial infarction
I24.0, I25.1, I25.2, I25.7, I25.8, I25.9	Known coronary artery disease
I27.0, I27.2	Chronic pulmonary hypertension
I70.2, I70.3, I70.5, I70.6, I70.7, I70.9	Peripheral vascular disease
I63, I65, I66	Ischemic cerebrovascular accident
Exclusion criteria codes	
I50.21, I50.31, I50.41, I50.811, I50.813	Isolated acute heart failure
02YA0Z0, 02YA0Z1, 02YA0Z2	Cardiac transplant
C0-C8, D0-D3	Malignancy
K90	Intestinal absorption disorders
E40, E41, E42	Rare nutritional disorders (i.e. kwashiorkor and marasmus)

Appendix 2 

**Table 5:** Propensity score matched groups (1:6) comparing PCM vs no-PCM baseline characteristics in chronic heart failure patients undergoing CABG or valve replacement cardiac surgeries from the NIS database between 2016 and 2018 in the United States. CABG: coronary artery bypass graft; CCI: Charlson Comorbidity Index; PCM: protein-calorie malnutrition; SD: standard deviation; NIS: National Inpatient Sample

Variables	CABG (N=1,729)			Valve replacement (N=4,018)		
	PCM (n=247,14.3%)	No PCM (n=1,482;85.7%)	p-value	PCM (n=574,14.2%)	No PCM (n=3,444; 85.7%)	p-value
Age, mean (SD)	68.8±9.3	68.7±9.0	0.86	74.6±12.6	75.0.7±12.1	0.45
Gender, n (%)						
Male	165 (66.8)	972 (65.6)	0.71	304 (53.0)	1821 (52.9)	0.97
Female	82 (34.4)	510 (33.2)		270 (47.0)	1623 (47.1)	
Race, n (%)						
White	174 (70.4)	1067 (72.0)	0.90	469 (81.7)	2806 (81.5)	0.91
Black	25 (10.1)	132 (8.9)		44 (7.7)	273 (7.9)	
Hispanic	22 (8.9)	121 (8.2)		18 (3.1)	92 (2.7)	
Other/missing	26 (10.5)	162 (10.9)		43 (7.5)	273 (7.9)	
CCI, n (%)						
Mild (1-2)	60 (24.3)	363 (24.5)	1.00	188 (32.8)	1129 (32.8)	0.98
Moderate (3-4)	78 (31.6)	465 (31.4)		224 (39.0)	1331 (38.6)	
Severe (>5)	109 (44.1)	654 (44.1)		162 (28.2)	984 (28.6)	
Primary payer, n (%)						
Medicare/medicaid	187 (75.7)	1136 (76.7)	0.77	478 (83.3)	2924 (84.9)	0.60
Private insurance	50 (20.2)	299 (20.2)		77 (13.4)	421 (12.2)	
Self-pay/other	10 (4.0)	47 (3.2)		19 (3.3)	99 (2.9)	
Hospital length of stay, mean (SD)	19.7±19.1 median: 14.0	10.0±9.6 median: 8.0	<0.001	13.2±13.5 median: 9.0	5.8±5.9 median: 4.0	<0.001
